# Readiness of health posts for primary health care integration in Indonesia: a mixed-methods study

**DOI:** 10.1186/s12889-025-22520-x

**Published:** 2025-04-16

**Authors:** Ririn Setiaasih, Deni Kurniadi Sunjaya, Yulia Sofiatin, Irvan Afriandi, Lukman Hilfi, Dewi Marhaeni Diah Herawati

**Affiliations:** 1 Master Program of Public Health, Faculty of Medicine, Universitas Padjadjaran, Bandung, Indonesia; 2 Department of Public Health, Faculty of Medicine, Universitas Padjadjaran, Bandung, Indonesia

**Keywords:** Health posts, PHC integration, Readiness, Health transformation

## Abstract

**Background:**

Primary Health Care Integration (PHCI) is part of Indonesia’s health transformation initiated in 2022. After two years, not all districts/cities have implemented the change, especially related to Health Posts (HP). The study aims to assess the HP’s readiness to implement this transformation.

**Method:**

A sequential exploratory mixed-method design was used. The first stage was explorative using a qualitative design and developing a quantitative research instrument. The second stage conducted a survey. Qualitative data collection through in-depth interviews and focus group discussions involving 42 informants: health cadres, health care workers, and City Health Office representatives. The qualitative data was processed with the ScreenQ software, and content analysis was carried out. Afterward, quantitative research instruments were built using predetermined theory, and validity and reliability tests were carried out. The quantitative survey involved 139 health cadres and 128 health care workers. The data obtained is transformed first using Rasch modeling (Winsteps 37). Then it was analyzed using SPSS software (SPSS 20), with readiness levels categorized into “not ready”, “moderately ready”, and “ready”. A comparison of readiness was performed between groups of respondents.

**Results:**

Qualitative analysis identified Facilitators including health cadres enthusiasm, collaboration, and commitment. Barriers included human resource, facilities, data synchronization, and digital reporting. Quantitative results showed that 83.5% of Health Posts were classified as “moderately ready”, meaning they had some essential elements in place but still faced gaps requiring further strengthening before full implementation could succeed. No statistically significant differences were found in readiness perceptions between health cadres and health care workers (3.19 logit, CI: -12.91–19.27, *p* = 0.697).

**Conclusion:**

This study provides baseline data for future research and policy development, offering insights into how to strengthen PHCI readiness in similar contexts, particularly by addressing the serious barriers that may hinder successful implementation.

**Supplementary Information:**

The online version contains supplementary material available at 10.1186/s12889-025-22520-x.

## Introduction

In the era of globalization, global health challenges have become increasingly complex, requiring countries, including Indonesia, to respond more effectively to demographic shifts, evolving disease patterns, and changing healthcare demands. To address these challenges, Indonesia has established a health service transformation policy aimed at building a more responsive healthcare system. As part of this transformation, Primary Health Care Integration (PHCI) ensures continuous, person-centered services across the life cycle while addressing health issues and social determinants. PHCI emphasizes public health promotion, disease prevention, and service integration [1].

The Indonesian government has enacted Law Number 17 of 2023 on Health as the legal foundation for strengthening healthcare services at various levels, reinforcing Indonesia’s commitment to adapting to global health challenges. This law also supports six key pillars of health transformation: primary healthcare services, referral systems, health system resilience, health financing, healthcare human resources, and health technology [1, 2].

The Primary Service Transformation is one of the six pillars of the health transformation, which includes the implementation of the PHCI strategy. The Ministry of Health emphasizes the provision of healthcare services throughout the life cycle, bringing services closer through village-level networks, and strengthening Local Area Monitoring through digitalization and home visits, applying the Healthy Indonesia Program with a Family Approach concept. Currently, there are 10,292 Community Health Centers (CHC) in Indonesia, but this number is insufficient to serve a population of 273.5 million people.

The distribution of CHCs is also uneven, with urban areas generally having better access to healthcare facilities compared to rural and remote regions. Many rural areas face limited health infrastructure, geographical barriers, and shortages of trained healthcare workers, further amplifying the urgency for strengthening village-level Health Posts (HP). The government aims to establish 300,000 HP at the village level to provide promotive and preventive services throughout the life cycle [3–5].

To support this transformation, lessons from other countries with successful community-based healthcare integration offer valuable insights. For instance, Brazil’s Family Health Strategy (ESF) has expanded primary healthcare coverage in urban areas, benefiting both registered populations and surrounding communities. However, its effectiveness depends heavily on the availability and capacity of healthcare teams, as well as the program’s success in addressing access disparities among vulnerable groups, including low-income populations and racial minorities. These international experiences underscore the importance of tailoring primary healthcare strategies to local contexts, ensuring that community-based approaches align with each population’s unique geographic, social, and cultural characteristics [6].

Thailand’s Village Health Volunteers (VHVs) program has also played a key role in strengthening community-level primary healthcare, particularly during the COVID-19 pandemic. Anwar et al. highlight that VHVs act as vital links between communities and health facilities, delivering health education, conducting contact tracing, and promoting disease prevention within their local areas. The program’s success stems from strong government support, ongoing capacity building, and a structured incentive system that sustains volunteer engagement. These findings reinforce the importance of empowering local volunteers with deep social and cultural understanding to enhance community acceptance of health programs and strengthen community resilience, both in urban and rural settings [7].

Inspired by these international models, Indonesia’s PHCI strategy aims to enhance the role of HP in delivering comprehensive, community-based services across all life stages. Saepudin’s research indicates that HP help facilitate community access to healthcare services and contribute to improving maternal and child health outcomes, while also changing public perceptions regarding healthcare-seeking behavior. Learning from the COVID-19 experience, health cadres in HP have proven to be important links between communities and the broader health system. Strengthening the capacity and competence of health cadres not only supports routine health services, but also serves as a sustainable investment in public health emergency preparedness, contributing to the development of a more resilient health system [8, 9].

Building upon these global insights, under the PHCI strategy, HP are envisioned as integrated village-level service units that provide comprehensive healthcare across all age groups—from early childhood to elderly care—ensuring service continuity and enhancing overall delivery. Health cadres are required to conduct home visits and master 25 core competencies related to healthcare services. This comprehensive approach is designed to ensure that all aspects of community health are effectively addressed, from preventive care to the management of various health conditions [10–12].

The theory underlying this research is using Sunjaya’s Readiness to engage theory, which is relevant in the context of measuring HP readiness to adopt PHCI. This theory identifies five dimensions that are important in evaluating the readiness of an entity or organization for change, namely awareness, comprehension, concern, involvement and support.16 Through this 5 (five) dimensional approach, this research is used to overcome the gap in understanding the practical implementation of PHCI at the Health Post level, which has so far only been systematically evaluated at the Community Health Center level. The aim of this research is to assess HP’s readiness to implement PHCI [13].

## Method

### Context of study

This research was conducted in a city in West Java Province with a population of 590,782 people. The city is located in the center of West Java and is divided into three sub-districts. The population is served by 13 community health centers (CHC) and two supporting CHC, with a CHC to population ratio of 1:42,183, which falls below the national standard of 1:30,000. The community participates through 402 Integrated HP spread across 15 sub districts [14, 15].

The selected city was chosen due to its ongoing primary healthcare transformation efforts, population diversity, and its relevance as a representative area facing typical challenges in implementing primary healthcare integration (PHCI). These challenges include limited health worker capacity, uneven community engagement, and resource constraints, making it a suitable setting for evaluating readiness to engage with PHCI.

### Study design

This study employs a sequential exploratory mixed-methods design, conducted in two phases: Phase 1 (qualitative) and Phase 2 (quantitative). The qualitative phase follows a constructivist paradigm, exploring how social interactions shape perceptions of readiness within Health Posts (HPs) for Primary Healthcare Integration (PHCI). Insights from this phase were used to develop a contextually relevant quantitative instrument. Meanwhile, the quantitative phase adopts a post-positivist paradigm, assessing HPs’ readiness to implement PHCI using validated instruments derived from qualitative findings [13, 16].

This mixed-methods approach ensures that the final assessment is both empirically robust and locally grounded, addressing the lack of standardized measurement tools in PHCI settings. The integration of qualitative and quantitative phases allows for a more comprehensive evaluation of readiness to engage, which is highly context-dependent. Data collection was conducted between May and June 2024 [13, 16].

### Phase 1: qualitative study

#### Data collection

The first phase utilized a qualitative approach to explore the readiness of healthcare personnel (HP) for PHCI implementation. Informants were selected through purposive sampling in collaboration with local CHCs and the city health office, ensuring participation from health cadres and healthcare workers actively involved in PHCI. Eligibility criteria included at least one year of work experience and direct involvement in PHCI.

Semi-structured interviews (30–60 min) were conducted using an interview guide based on Readiness to Engage Theory (see Supplementary Materials). Triangulation was applied by cross-referencing interviews with document reviews (local health policies and PHCI guidelines) and focus group discussions (FGDs) with senior health officials. Member checking was performed by summarizing preliminary findings and obtaining participant feedback to enhance validity [17, 18].

#### Data analysis

In the qualitative phase, data were analyzed using ScreenQ, a computer-assisted qualitative data analysis software (CAQDAS) designed to enhance transparency and rigor through systematic coding, categorization, and thematic development. Unlike manual coding, ScreenQ facilitates structured data management, allowing researchers to systematically apply and refine coding frameworks while maintaining an audit trail of coding decisions, thereby improving transparency and reproducibility. The software enabled the application of a pre-defined theoretical framework (Readiness to Engage Theory) for initial coding while also allowing for inductive identification of new themes, ensuring both theoretical consistency and openness to emerging insights [17, 18].

A thematic analysis was conducted to identify key dimensions of HP readiness for PHCI implementation, which were subsequently translated into measurable survey items for the quantitative phase. To enhance the validity of findings, triangulation methods—combining interviews, focus group discussions, and document reviews—were employed to minimize researcher bias. Member checking further validated the interpreted themes, strengthening the trustworthiness of the results.

Findings from Phase 1 directly informed the development of the Phase 2 survey instrument, ensuring that it captured locally relevant dimensions of readiness to engage with PHCI [17, 18].

### Phase 2: quantitative study

#### Data collection

The second phase employed a cross-sectional survey to measure readiness to engage with PHCI. The survey instrument was developed by operationalizing key qualitative themes into structured survey items. The preliminary version of the questionnaire underwent a pilot test with 10 healthcare workers to assess clarity, relevance, and comprehensiveness. Based on feedback, minor revisions were made to improve item wording.

The final questionnaire was distributed to 139 health cadres and 128 healthcare workers across 12 CHCs in three sub-districts. Stratified sampling was applied to ensure representation across sub-districts, with proportional allocation based on workforce distribution. The English version of the questionnaire is provided as a supplementary file [17, 19, 20].

#### Data analysis

In the quantitative phase, statistical analyses were performed to evaluate the instrument’s validity and reliability and to compare readiness levels between health cadres and healthcare workers using an independent T-test.

The Rasch model was employed to assess instrument properties, including unidimensionality, item fit statistics, person and item reliability, and item difficulty calibration. Items with misfit statistics outside acceptable thresholds were revised or removed, ensuring that the final instrument retained strong psychometric properties. This iterative refinement process ensured the instrument was both contextually relevant and statistically robust, accurately capturing health workers’ readiness to engage with PHCI.

The final instrument demonstrated high reliability and strong measurement properties, with Item Reliability = 0.98, Item Separation = 7.95, Cronbach’s Alpha = 0.88, Raw Variance Explained by Measure = 34.9%, and Unexplained Variance in the 1st Contrast = 12.3% [21–23].

### Readiness categorization

Readiness levels were categorized based on Rasch person measure scores, which were mapped into three distinct levels: ‘Not Ready’ (below − 1 standard deviation), ‘Moderately Ready’ (between − 1 and + 1 standard deviation), and ‘Ready’ (above + 1 standard deviation). This approach, commonly applied in Rasch analysis, allows for the classification of respondents along a continuous readiness spectrum, ensuring that the resulting categories are empirically grounded in the actual data distribution rather than relying on arbitrary cut-off points [24, 25].

### Ethical consideration

The study adhered to ethical standards, ensuring informed consent from all participants, confidentiality of data, and approval from the relevant ethical review board was approved on May 14, 2024 Number 524/UN6.KEP/EC/2024 by the Ethics Committee of Universitas Padjadjaran.

## Results

### Qualitative results

The findings of this phase are categorized into several main predetermined themes, including awareness, comprehension, concern, involvement, and support. Two other constructs were generated: facilitators and barriers. The following section presents Table 1, which outlines 12 sub-themes within the seven main themes, providing a comprehensive overview of the qualitative findings. The characteristics of the informants supporting this understanding are detailed in Table 2, which provides further information on their demographic and professional distribution.

**Table 1:** Themes and Sub-themes identified from the qualitative analysis

Theme	Sub-theme
Awareness	Knowledge of PHCI
	Training
	Socialization
	Source of Information
Comprehension	Understanding of PHCI Objectives
	Scope of PHCI
	Integration of Services
	Competency Mastery
Concern	Burden on Health Cadres
	Increased Workload
	Service Quality
	Service Integration
Involvement	Health Care Workers
	Education
	Reporting
	Health Cadres
	Promotion Activities
	Dedication
Support	Health Office
	Adaptability
	Local Government
	Role of Sub-district
Facilitators	Health cadres Support
	Enthusiasm
	Collaborative Support
	Cooperation
	Flexibility
	Local Needs
Barriers	Implementation Challenges
	Human Resources
	Facilities
	Data Synchronization
	Digital Reporting

The following sections provide a detailed exploration of each theme.

### Awareness

The informants demonstrated varying levels of understanding about PHCI. Most informants had a good grasp of the concept. SU, a health care worker, explained:

*We’ve actually been aware of PHCI since last year and only recently completed training provided by City Health Office*,* (SU*,* healthworker)*

DA mentioned receiving information during meetings at the City Health Office. Likewise, FA and RA noted that they gained information through socialization efforts by the Ministry of Health and Health Office. LA, another health care worker, added:

*I first learned about PHCI through a webinar hosted by the Ministry of Health*,* and later from in-person outreach conducted by the Provincial Health Office and City Health Office. (LA*,* health care worker)*

However, some informants, like VN and SE, noted that despite these efforts, knowledge about PHCI was not uniformly distributed among the cadres. VN, a health care worker, observed:

*Most of us learned about PHCI through the Ministry of Health’s webinar. The outreach from the City Health Office has mostly reached those involved in health promotion*,* nutrition programs*,* and Adult and Elderly HP. (VN*,* health care worker)*

Similarly, SE, a health care worker, noted:

*I only recently became aware of PHCI*,* as part of an initial introduction. (SE*,* health care worker)*

### Comprehension

Informants displayed a solid understanding of PHCI’s policies and objectives. RO, a City Health Office staff member, explained:

*PHCI serves the community across all age groups*,* from pregnancy to the elderly. (RO*,* City Health Office Staff)*

DA highlighted the goal of PHCI:

*PHCI to ensure that activities in primary services are integrated with each other. (DA*,* City Health Office staff)*

FA emphasized PHCI’s role in improving service quality but pointed out challenges, particularly the requirement for health cadres to master 25 competencies and integrate multiple services:

*PHCI is very important*,* because it can help provide health services to the community. In this PHCI*,* all health cadres must master 25 basic competencies (FA*,* health care worker)*

### Concern

One of the primary concerns raised by informants was the increased burden on health cadres, particularly the requirement to master a wide range of competencies and provide services across all life stages. IR noted:

*PHCI is actually very good*,* but it will be tough on the health cadres if all life cycles have to be served together. Health cadres need to be able to handle everything*,* mean while they are getting older. (IR*,* health care worker)*

Another concern was the potential decline in service quality when combining multiple age groups into a single integrated setting. RA stated:

*The concern of PHCI might be the quality of health services if multiple age groups are served simultaneously*,* (RA*,* health care worker)*

This highlights challenges in maintaining service standards, particularly since cadres typically specialize in specific age groups.

### Involvement

Healthcare workers and health cadres play distinct yet complementary roles in PHCI implementation. Healthcare workers primarily contribute by training health cadres, managing data reporting, and conducting home visits. YK stated:

*I am involved in basic competency training for the health cadres (YK*,* health care worker)*

SN highlighted their role in supporting cadres:

*Reporting has been good so far. Moreover*,* they are also willing to participate in home visits when requested*,* (SN*,* health care worker)*

*As health care workers*,* we frequently help with reporting tasks*,* even though it’s the health cadre’s responsibility*,* because many of them struggle with the digital reporting system. (SN*,* health care worker)*

Health cadres, on the other hand, are actively engaged in promoting PHCI and disseminating information. RN shared:

*I actively participate in health promotion activities*,* including spreading awareness about the PHCI program. (RN*,* health cadre)*

RA observed that health cadres exhibit strong commitment:

*The health cadres have shown remarkable enthusiasm for PHCI*,* which reflects their strong dedication to the program. (RA*,* health care worker)*

### Support

Support from various stakeholders, including the City Health Office and local government, was identified as a significant enabler of PHCI implementation. IR emphasized the role of training:

*Next week*,* we have a one-week PHCI training for all healthcare workers*,* provided by the Health Office. (IR*,* health care worker)*

Collaboration with local government was also recognized as crucial. VN mentioned:

*This morning*,* we attended meeting with the Local Government*, *to discuss the PHCI implementation plan. (VN*,* healthworker)*

### Facilitators

Several key factors were identified as facilitators in PHCI implementation:

- Strong morale among health cadres, JU noted:

*Health cadres have shown strong enthusiasm for all health programs*,* including PHCI*,* and this must be maintained. (JU*,* health cadre)*

- Collaboration among stakeholders, RN emphasized:

*Effective PHCI implementation requires strong cooperation among all involved parties to ensure resource sharing and aligned efforts. (RN*,* health cadre)*

- Flexibility in implementation, AW stated:

*It must be flexible… It doesn’t have to be implemented in a rigid way; what matters is achieving PHCI’s goal of providing healthcare across the entire life cycle. (AW*,* health care worker)*

- Adaptation to local conditions, RO highlighted:

*Considering the city’s conditions*,* PHCI must be adaptable. The key is ensuring that all populations receive services. (RO*,* City Health Office Staff)*

### Barriers

Despite strong support, several barriers were identified:

- Shortage of human resources and limited facilities, NW noted:

*We face two main challenges: the number and capability of health cadres*,* and the availability of adequate space in HP*,* which must accommodate services for multiple age groups simultaneously*,* increasing visitor numbers. (NW*,* health cadre)*

- Inadequate service locations, SN observed::

*The head of the neighborhood association often offers their house for PH activities*,* but due to the high number of visitors*,* this poses a risk. (SN*,* health care worker)*

- Educational disparities among health cadres, RR stated:

*The various educational backgrounds of health cadres might complicate their mastery of the 25 required competencies. (RR*,* health care worker)*

- Limited number of cadres and overlapping roles, EG explained:

*Some HP centers have only four or five health cadres. Additionally*,* some cadres handle both HP and Adult & Elderly HP*,* resulting in alternating service days. (EG*,* health cadre)*

- Challenges in digital reporting, DN and IR described difficulties:

*Currently*,* Adult and Elderly HP and HP data are separate. If not updated*,* residents may only see one set of HP data. (DN*,* healthworker)*

*Since we are in the digital era*,* reporting is done via links. Sometimes*,* we’re asked to fill out multiple links*,* which can be confusing. It’s easy to feel overwhelmed. (IR*,* health care worker)*

- Limited service spaces, EG added:

*The available spaces are only sufficient for conducting HP activities alternately. (EG*,* health cadre)*

### Quantitative results

The quantitative analysis included 139 health cadres and 128 health care workers, with a clear gender disparity. Males represent only 6.0% (15) of the sample, whereas females make up 94.4% (252). Subjects were selected with careful consideration to ensure representation from three districts within the city. Table 1 details their demographic and professional attributes. These characteristics are crucial for understanding the data’s context and ensuring the sample’s representativeness in relation to the study’s overall objectives, contributing to a more comprehensive analysis.

Table 2 highlights notable trends, such as the predominance of female respondents and the significant proportion of professionals holding higher education degrees, underscoring the generally well-educated workforce involved in this study. Regarding age, most respondents (52.8%, 141) were between 41 and 60 years old. The majority comprised health cadres (52.1%, 139), with a significant proportion holding a bachelor’s degree (50.9%, 136). Additionally, 65.9% (176) of the respondents reported having 1–10 years of professional experience, suggesting a workforce with moderate levels of experience in their respective roles.

**Table 2:** Characteristics of respondents

	Qualitative		Quantitative	
	*n* = 42	%	*n* = 267	%
Gender				
Male	2	4.8	15	6.0
Female	40	95.2	252	94.4
**Age (year)**				
21–40	16	38.1	113	42.3
41–60	25	59.5	141	52.8
> 60	1	2.4	13	4.9
**Profession**				
Health cadres	26	61.9	139	52.1
Health care worker	14	33.3	128	47.9
City Health Office Staff	2	4.8	0	0
**Education Level**				
Elementary School	1	2.4	10	3.7
Junior High School	6	14.3	35	13.1
Senior High School	18	42.9	86	32.2
Bachelor’s Degree	17	40.5	136	50.9
**Years of work experience**				
1–10	28	66.7	176	65.9
> 10	14	333	91	34.1

**Fig. 1:**
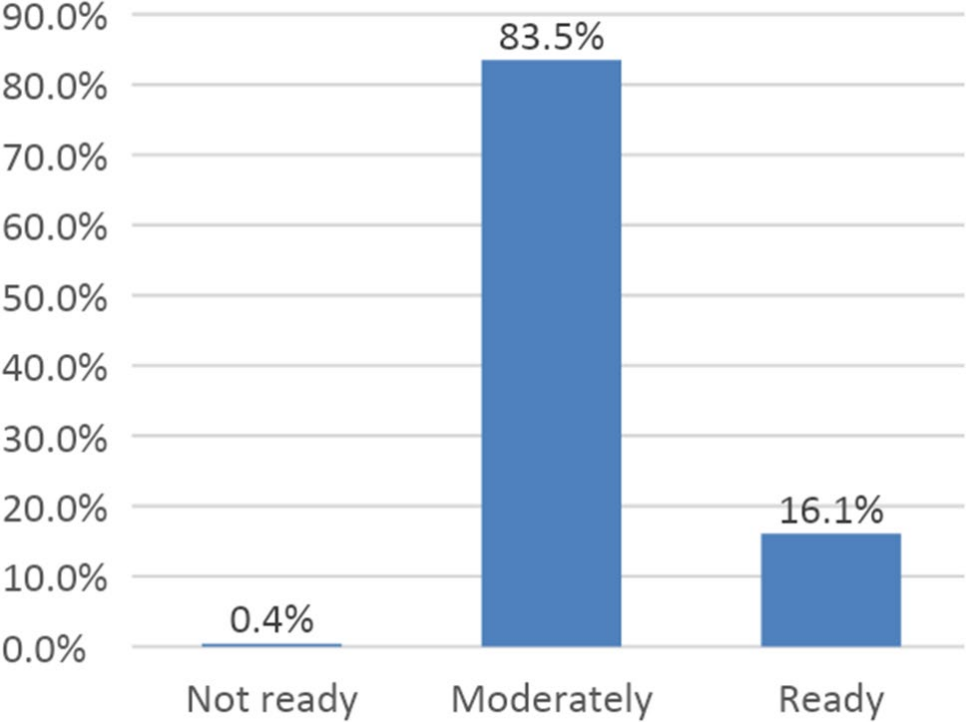
Health posts readiness distribution

Figure 1 presents the distribution of HP readiness levels to implement Primary Healthcare Integration (PHCI), as assessed by respondents and analyzed using Rasch. Categories include ‘not ready’ (below − 1SD), ‘moderately ready’ (between − 1SD and + 1SD), and ‘ready’ (above + 1SD). The majority of respondents (83.5%) rated their HPs as ‘moderately ready,’ while 16.1% assessed them as ‘ready,’ and only 0.4% considered them ‘not ready.’ This distribution underscores that while most HPs demonstrate adequate preparedness, targeted interventions may be required to elevate readiness across all centers for successful PHCI implementation.

**Fig. 2:**
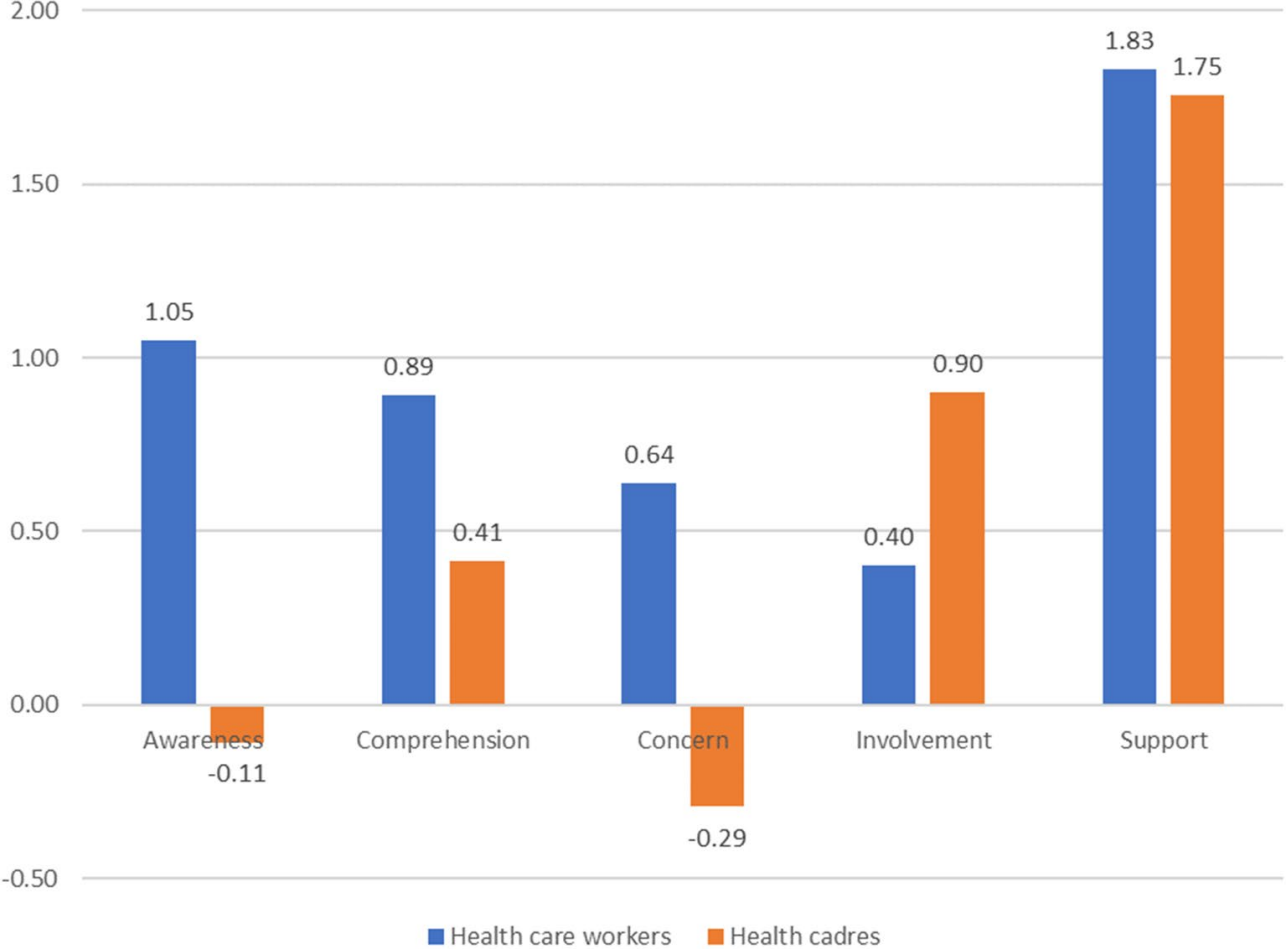
Readiness dimensions: Health care workers vs. Health cadres

Figure 2 compares health cadres and healthcare workers across five dimensions of the Readiness to Engage theory. Healthcare workers scored higher in awareness (1.05 vs. -0.11), comprehension (0.89 vs. 0.41), and concern (0.64 vs. -0.29), reflecting their stronger alignment with program objectives. In contrast, health cadres excelled in involvement (0.90 vs. 0.40), emphasizing their active participation at the community level. Both groups reported similar levels of support, with minimal differences (1.83 vs. 1.75), indicating consistent resource availability for readiness initiatives.

Table 3 presents the results of the independent samples t-test, which indicate no statistically significant difference in perceptions between health cadres and healthcare workers (*p* = 0.697). Both groups demonstrated comparable readiness levels. Standard deviation values were relatively high (73.17 for health care workers and 60.15 for health cadres), indicating considerable variability within each group.

**Table 3:** Comparison of readiness scores between health care workers and health cadres

Group	*N*	Mean	Std.Dev.	Mean Difference	t	df	*p*-value	95% Confidence Interval
Health care workers	128	57.96	73.17	3.19	0.39	265	0.697	− 12.91–19.27
Health cadres	139	54.77	60.15					

### Triangulation analysis narrative (Readiness to Engage)

The triangulation analysis on readiness to engage in PHCI implementation, summerized in Table 4 reveals a strong convergence across five key themes: awareness, comprehension, concern, involvement, and support.

Both qualitative and quantitative findings consistently show that health care workers demonstrate higher awareness and comprehension of PHCI compared to health cadres, indicating the need for improved dissemination of information at the community level. In terms of concern, health care workers are primarily focused on policy-level and cross-sectoral collaboration challenges, whereas health cadres express more operational and logistical concerns related to their fieldwork responsibilities. This highlights the importance of addressing both strategic and practical barriers simultaneously.

**Table 4:** Convergence and divergence analysis (Five themes of readiness to Engage)

Theme	Qualitative Findings	Quantitative Findings	Convergent / Divergent	Explanation
Awareness	Informants stated that both health workers and community health health cadres (kader) had heard about PHCI, mainly through socialization activities and webinars. However, their detailed understanding of its goals and benefits remained limited, especially among the health cadres	Awareness scores: - Health workers: 1.05 - Health cadres: -0.11	Convergent	Both data sources indicate that health workers have higher awareness and understanding of PHCI compared to health cadres
Comprehension	Some health workers reported having a general understanding of PHCI, but they felt they had not received clear technical guidance. health cadres mentioned they were only familiar with the term, without fully understanding the practical details of its implementation	Comprehension scores: - Health workers: 0.89 - Health cadres: 0.41	Convergent	Both sources show that health workers have better comprehension than health cadres, although overall comprehension is still weak
Concern	Health workers expressed concerns about limited resources, particularly regarding cross-sectoral coordination. health cadres were more concerned about technical challenges in the field, such as funding and limited facilities	Concern scores: - Health workers: 0.64 - Health cadres: -0.29	Convergent	Both groups show concerns, although their focus differs: health workers worry about coordination, while health cadres focus on operational issues
Involvement	Health cadres reported being more involved in community outreach and assisting residents, while health workers were more engaged in cross-sectoral coordination and program planning	Involvement scores: - Health workers: 0.40 - Health cadres: 0.90	Convergent	Both data sources confirm that health cadres play a more active role in field activities compared to health workers
Comprehension	Some health workers reported having a general understanding of PHCI, but they felt they had not received clear technical guidance. health cadres mentioned they were only familiar with the term, without fully understanding the practical details of its implementation	Comprehension scores: - Health workers: 0.89 - Health cadres: 0.41	Convergent	Both sources show that health workers have better comprehension than health cadres, although overall comprehension is still weak
Support	Informants stated that facilities and policy support from the health office and local government were available. However, monitoring and evaluation of PHCI implementation were considered insufficient	Support Scores: - Health workers: 1.83 - Health cadres: 1.75	Convergent	Both groups agree that support is available, but there are still gaps in monitoring and evaluation processes

Regarding involvement, health cadres play a more prominent role in direct community engagement, while health care workers are more involved in technical coordination and program oversight. This complementary division of roles is consistently reflected in both datasets. Lastly, both data sources highlight the availability of policy and facility-level support, but also point to weaknesses in monitoring and evaluation mechanisms. These convergent findings emphasize the need to strengthen performance tracking and continuous improvement efforts for PHCI implementation.

Overall, the strong alignment between qualitative and quantitative findings enhances the credibility of the results, providing a comprehensive and validated understanding of the readiness to engage across different stakeholder groups. This integrated perspective can inform the design of targeted interventions to improve both technical capacity and community-level participation in PHCI programs.

## Discussion

The lack of statistically significant differences between health cadres and health care workers, despite observable differences in mean scores across some dimensions, suggests that these variations may stem from random fluctuations rather than systematic differences between the groups. The relatively large standard deviation values observed for both groups (73.17 for health care workers and 60.15 for health cadres) indicate substantial variability in individual responses, suggesting the need for deeper examination of underlying factors contributing to these differences.

This high variability could be attributed to the heterogeneous characteristics of respondents, as shown in Table 2. Factors such as wide-ranging ages, diverse educational backgrounds, and varying lengths of professional experience may contribute to differing levels of understanding, engagement, and readiness to implement Primary Healthcare Integration (PHCI). For instance, younger respondents with limited experience may perceive readiness differently compared to their more experienced counterparts, while those with higher educational attainment might demonstrate greater comprehension and awareness of the program’s objectives [26, 27].

This observed variability highlights the importance of tailoring readiness-enhancing interventions to address the specific needs of diverse subgroups within the health workforce, rather than assuming uniform perceptions and capabilities across all cadres and health care workers [26].

### Awareness and comprehension

Understanding of how to apply PHCI varied among respondents, despite the fact that this information was delivered through training by the Health District Office. This result highlights the need to improve strategies for disseminating consistent and comprehensive information to all relevant parties, especially health cadres. This condition is not specific to PHCI; a study on tuberculosis case detection by Sunjaya in West Java showed that systemic and structural factors influence how information and support are delivered to various levels of health care workers [13].

However, this study also reveals significant differences in readiness levels between health care workers and health cadres in implementing PHCI, with health care workers exhibiting higher awareness and comprehension. This discrepancy is likely due to the more intensive training and better access to information that health care workers receive, a factor not observed in Sunjaya’s study. To address this gap, enhanced training and educational materials for health cadres are necessary, as supported by Naidoo’s research on the importance of training in adapting to changes in healthcare services [28].

### Concern

The findings reveal a significant difference in concern levels between health care workers and health cadres regarding the implementation of PHCI. Health care workers exhibit higher concern levels, it is likely due to more intensive training and better access to information. Health cadres, however, express concerns about the increased workload and potential impact on healthcare service quality due to consolidation. These concerns align with Sunjaya’s study, which reported those who are not involved in the official program, such as private practice in tuberculosis transmission risk program, faced similar challenges due to cost and limited resources. Those challenges affecting their active participation in following and reporting the program [13].

Additionally, HP in Indonesia often face challenges such as inadequate health cadres training and support, which can hinder their performance and, consequently, affect the public’s willingness to utilize HP services. While efforts to empower health cadres through training and discussions improve knowledge and skills, continued support and monitoring from CHC are needed to ensure the optimal function of HP. The similarity in concerns across these studies indicates that perceived threats, such as health risks, increased workload, and suboptimal functioning of HP, can hinder an individual’s readiness to engage in new health programs. Therefore, it is crucial to provide additional support, resources, and sufficient training to alleviate workload pressures, improve service quality, and ensure the readiness of all involved in the implementation of PHCI or other health programs [29].

### Involvement

This study found that the level of involvement of health cadres in the management of HP is higher compared to health care workers. This finding aligns with A’la’s scoping review research, which identifies that the concept of involvement, particularly in the context of volunteering in the health sector, is a complex attribute and often higher among individuals motivated by social and philanthropic factors. A’la also emphasizes that volunteer involvement, such as that of the health cadres, has significant consequences both for the individuals and the healthcare system as a whole. Therefore, the higher involvement of health cadres in this study may be influenced by stronger intrinsic motivation and a sense of social responsibility, contributing positively to the effectiveness of HP services [30].

Furthermore, Ridwan’s research supports these findings by showing that certain factors are associated with the activeness of health cadres, such as age, education, knowledge, completeness of facilities, and the role of health care workers. Ridwan found that all these factors have a significant relationship with the activeness of health cadres, reinforcing the conclusion that more involved health cadres tend to have higher motivation, supported by adequate knowledge and facilities. These findings further strengthen the argument that high health cadres involvement in HP management is crucial for the success of the program and for improving the quality of healthcare services in the community [31].

### Support

This study underscores the pivotal role of support from various parties, including the city health office and local government, in ensuring the successful implementation of PHCI. Effective training, collaboration, and stakeholder engagement are critical factors for the program’s success. These findings align with Mauco’s research in Botswana, which emphasizes the importance of a comprehensive e-health readiness framework for developing countries. Mauco’s study highlights the need for assessing both technical and non-technical readiness and engaging stakeholders, reflecting similar themes of support and resource adequacy found in this study [32].

Additionally, the results are comparable to those from Nyalunga’s research on health cadres in South Africa. Nyalunga’s study revealed that despite facing challenges such as limited equipment and safety concerns, most CHWs had positive perceptions of their training, teamwork, and practice. This suggests that, similar to the findings in this study, effective support and proper training are essential for overcoming obstacles and enhancing the performance of health programs. Both studies highlight that while challenges remain, the availability of resources and support can significantly impact the success and efficiency of health initiatives [33].

### Policy recommendations for PHCI readiness

To enhance the readiness of health cadres and health care workers for PHCI implementation, several key strategies can be recommended, based on the study findings and supported by previous research.

1. Targeted and Tiered Training Programs
  Routine PHCI training should not only emphasize practical applications but also be adapted to the educational background and prior experience of both health cadres and health care workers. This aligns with Ridwan et al.’s recommendations in Jambi, which highlighted the importance of practical and context-specific training [31]. Health cadres with lower educational attainment should receive more hands-on, foundational training, while health care workers could benefit from advanced modules focusing on coordination, evaluation, and multi-sectoral collaboration. Regularly updated training content will ensure relevance and address evolving program needs.
2. Performance-Based and Non-Monetary Incentives
  Clarifying the benefits of PHCI and providing performance-linked, non-monetary incentives can enhance motivation and foster stronger commitment among health cadres. As Profita’s research suggests, motivation and support play crucial roles in sustaining engagement and readiness [34]. Local governments could allocate specific budgets to support incentives such as phone credit for outreach, transportation for home visits, and community recognition awards. These incentives should be linked to clear performance indicators, such as attendance at training, timely reporting, and active participation in PHCI-related activities.
3. Strengthened Supervision and Mentoring Mechanisms
  Effective supervision and ongoing mentoring are essential to sustain health cadres’ motivation and ensure consistent program implementation. CHC should establish regular mentoring sessions—ideally monthly—where health cadres and health care workers collaboratively review progress, share challenges, and receive technical updates. This forum would not only clarify roles and expectations but also serve as a platform for collaborative problem-solving and peer learning, ultimately fostering a sense of shared ownership over PHCI success.
4. Cross-Sectoral Coordination and Integration
  Subdistrict-level coordination forums should be revitalized to strengthen cross-sectoral collaboration. Integrating PHCI into broader health and social programs can enhance synergy, prevent duplication, and ensure health cadres and health care workers have clear information on available resources and referral pathways. Effective cross-sectoral coordination has been shown to improve program coverage and sustainability [35].
5. Community Empowerment and Awareness Campaigns
  Community awareness and involvement are critical for sustaining health cadres’ motivation and increasing community support for PHCI. In collaboration with village governments, the District Health Office could launch community campaigns to enhance public understanding of PHCI objectives and benefits. Increased community awareness could improve the social standing of health cadres, reinforcing their motivation and sense of purpose. This aligns with Nyalunga et al.’s findings, which highlight how community recognition and support contribute to positive perceptions of health workers’ roles and training [33].

### Final emphasis

Ultimately, improving PHCI readiness requires a comprehensive approach that combines capacity building, ongoing support, performance-based incentives, and stronger coordination between health facilities, local governments, and communities. These efforts must also account for contextual factors, such as target population size, available infrastructure, and overall support systems, as highlighted by Ridwan et al. [31, 34].

## Limitation of the study

This study has several limitations. First, it relies on self-reported data, which may introduce bias and inaccuracies, including social desirability bias, where respondents might provide answers they perceive as favorable. Second, the gender imbalance among respondents could limit the generalizability of the findings. However, this distribution reflects the actual demographic composition of health cadres in Indonesia, where the majority are women. Third, the study includes participants with diverse educational backgrounds, which may have influenced their understanding and interpretation of the PHCI implementation process, leading to potential variations in responses. Lastly, since the study was conducted in a single city in West Java, the findings may not be fully applicable to other regions. Differences in socio-economic conditions and healthcare infrastructure across Indonesia highlight the need for further research to validate these findings in broader contexts.

## Strength of the study

The strength of this study lies in its use of a sequential exploratory mixed-method design, combining qualitative and quantitative approaches to provide an in-depth understanding of the readiness of HP to implement Primary Health Care Integration (PHCI). The study involved relevant informants, including health cadres, health care workers, and representatives from the City Health Office, and employed data triangulation to enhance the validity of the qualitative findings. Additionally, the development of a quantitative instrument based on the readiness to engage theory has been tested for validity and reliability, ensuring trustworthy data. The application of Rasch analysis to the quantitative data further improved the quality of instrument analysis. This research provides important baseline data on PHCI implementation readiness and can serve as a reference for health policy development and future research.

## Conclusions

This study assessed the readiness of Health Posts (HP) for the implementation of Primary Health Care Integration (PHCI) within Indonesia’s health transformation initiative, focusing on one city in West Java. The findings indicate that most Health Posts in this context are moderately ready, meaning they have some essential elements in place. However, this moderate readiness exists alongside serious challenges that could hinder successful implementation if not adequately addressed. These challenges include significant limitations in human resources, insufficient facilities, and persistent issues related to data synchronization and digital reporting.

Key facilitators identified include health cadres’ enthusiasm, effective collaboration, and strong stakeholder commitment, but these positive factors are often offset by the structural and operational weaknesses observed during the study. No significant differences were found in readiness perceptions between health cadres and health care workers, suggesting alignment in their views on the current state of readiness. These results provide baseline data that can inform future research and targeted interventions to address the identified gaps, particularly in similar urban settings, while recognizing that readiness levels may vary across different regions with diverse health system capacities.

## Electronic supplementary material

Below is the link to the electronic supplementary material.

Supplementary Material 1

Supplementary Material 2

Supplementary Material 3

Supplementary Material 4

Supplementary Material 5

Supplementary Material 6

## Data Availability

The English version of the questionnaire used in this study is available as a supplementary file in this manuscript. Data supporting the findings of this study are available upon request from the corresponding author.
