# Supplementary material for: Readiness of health posts for primary health care integration in Indonesia: a mixed-methods study
Source: BMC Public Health. 2025 Apr 16;25:1429. doi: 10.1186/s12889-025-22520-x (PMC12001397; doi:10.1186/s12889-025-22520-x)
Supplement: Supplementary file 1 — Supplementary Material 1 [file 12889_2025_22520_MOESM1_ESM.pdf]

Independent T-Test Output from SPSS

T-Test

[DataSet1]

Group Statistics

	profesi	N	Mean	Std. Deviation	Std. Error Mean
logit	1	128	57.9531	73.17016	6.46739
	2	139	54.7698	60.15491	5.10227

Independent Samples Test

		Levene's Test for Equality of Variances		t-test for Equality of Means						
									95% Confidence Interval of the Difference	
		F	Sig.	t	df	Sig. (2-tailed)	Mean Difference	Std. Error Difference	Lower	Upper
logit	Equal variances assumed	1.812	.179	.390	265	.697	3.18334	8.17211	-12.90719	19.27387
	Equal variances not assumed			.386	246.433	.700	3.18334	8.23774	-13.04201	19.40869
