# Supplementary material for: Readiness of health posts for primary health care integration in Indonesia: a mixed-methods study
Source: BMC Public Health. 2025 Apr 16;25:1429. doi: 10.1186/s12889-025-22520-x (PMC12001397; doi:10.1186/s12889-025-22520-x)
Supplement: Supplementary file 2 — Supplementary Material 2 [file 12889_2025_22520_MOESM2_ESM.pdf]

Qualitative Questionnaire on Health Posts Readiness in Implementing Primary Health Care Integration (PHCI) for City Health Office Staff

RESPONDENT INFORMATION

Gender:

- Male
- Female

Age:

- 21-40
- 41-60
- > 60

Profession:

- Health Worker
- Cadre
- City Health Office Staff

Education Level:

- Elementary School
- Junior High School
- Senior High School
- Bachelor's Degree

Years of work experience:

- 1-10
- > 10 years

QUESTIONNAIRE

1. Awareness

- Have you heard about the Transformation of Primary Health Services (PHCI)? Where did you get the information from?
- Before PHCI was implemented, what activities were conducted at the Health Post to improve public health?
- How do you introduce the concept of PHCI to the health cadres at the Health Post?
- Do you think everyone at the Health Post is aware of PHCI? How do you know?
- Do you know why PHCI is necessary?

2. Comprehension

- Do you understand what PHCI is and its objectives?
- Can you briefly explain the concept of PHCI at the Health Post?
- How do you think the health cadres at the Health Post understand PHCI?
- Are there any who are still confused among them?
- Have you noticed any changes in the services at the Health Post since PHCI was implemented?

3. Concern

- How important do you think PHCI is?
- Do you feel that PHCI will help many people?

- How do you ensure that the health cadres at the Health Post are also concerned and supportive of PHCI?
- Do you have any specific concerns regarding the implementation of PHCI at the Health Post?

4. Involvement

- Are the health cadres at the Health Post involved in the PHCI plan? What roles do they play?
- How do you involve everyone at the Health Post to implement PHCI?
- Have you been actively involved in the change process at the Health Post since PHCI was introduced?

5. Support

- What have you done to support PHCI at the Health Post?
- Do you feel that the support from relevant parties is sufficient to support PHCI at the Health Post?

6. Supporting and Inhibiting Factors

- From your experience, what factors have been most helpful in the implementation of PHCI at the Health Post?
- Have you received any support or assistance from other parties to help implement PHCI at the Health Post?
- What are the main obstacles you have faced in implementing PHCI at the Health Post?
- How have you or your team responded to these obstacles to ensure the smooth implementation of PHCI at the Health Post?
