# Supplementary material for: Readiness of health posts for primary health care integration in Indonesia: a mixed-methods study
Source: BMC Public Health. 2025 Apr 16;25:1429. doi: 10.1186/s12889-025-22520-x (PMC12001397; doi:10.1186/s12889-025-22520-x)
Supplement: Supplementary file 3 — Supplementary Material 3 [file 12889_2025_22520_MOESM3_ESM.pdf]

Qualitative Questionnaire on Health Posts Readiness
in Implementing Primary Health Care Integration (PHCI) for Health Cadres

RESPONDENT INFORMATION
Gender:
- Male
- Female

Age:
- 21-40
- 41-60
- > 60

Profession:
- Health Worker
- Cadre
- City Health Office Staff

Education Level:
- Elementary School
- Junior High School
- Senior High School
- Bachelor's Degree

Years of work experience:
- 1-10
- > 10 years

QUESTIONNAIRE

1. Awareness

- Have you heard about the Transformation of Primary Health Services (PHCI) at the Health Post?
- Where did you get the information?
- Before the PHCI was implemented, what activities were conducted at the Health Post to improve public health?
- Have there been any communication efforts from the City Health Office or health workers from the Community Health Centers (CHC) to inform the health cadres about PHCI?
- Is everyone at the Health Post aware of PHCI? How do you know?
- Do you know why PHCI is necessary?

2. Comprehension

- Do you clearly understand what changes have been made in PHCI and its objectives?
- Can you briefly explain the concept of PHCI at the Health Post?
- How do you think the health cadres at the Health Post understand PHCI?
- Have there been any difficulties in understanding PHCI?
- Have there been any competency training sessions provided to the health cadres at the Health Post?

3. Concern

- How important do you think the implementation of PHCI is at the Health Post?

- Do you believe PHCI will significantly benefit the community?
- How do you ensure other health cadres at the Health Post are also concerned and supportive of PHCI?
- Do you have any particular concerns about the implementation of PHCI at the Health Post?

4. Involvement

- Are you involved in the planning and implementation of PHCI at the Health Post? If so, what is your role in this process?
- How do you involve everyone at the Health Post to implement PHCI?
- To what extent have you actively participated in the change process at the Health Post since PHCI was introduced?

5. Support

- What have you done to support the PHCI plan at the Health Post?
- What is your view on the support provided by the City Health Office in supporting PHCI at the Health Post?
- Do you think this support is sufficient?

6. Supporting and Inhibiting Factors

- From your experience, what factors have been most helpful in the implementation of PHCI at the Health Post?
- Have you received any support or assistance from other parties to help implement PHCI at the Health Post?
- What are the main obstacles you have encountered in implementing PHCI at the Health Post?
- How have you or your team responded to these obstacles to ensure the smooth implementation of PHCI at the Health Post?
