# Supplementary material for: Readiness of health posts for primary health care integration in Indonesia: a mixed-methods study
Source: BMC Public Health. 2025 Apr 16;25:1429. doi: 10.1186/s12889-025-22520-x (PMC12001397; doi:10.1186/s12889-025-22520-x)
Supplement: Supplementary file 5 — Supplementary Material 5 [file 12889_2025_22520_MOESM5_ESM.pdf]

Quantitative Questionnaire on Health Posts Readiness
in Implementing Primary Health Care Integration (PHCI)

RESPONDENT INFORMATION

Gender:

- Male
- Female

Age:

- 21-40
- 41-60
- > 60

Profession:

- Health Worker
- Cadre

Education Level:

- Elementary School
- Junior High School
- Senior High School
- Bachelor's Degree

Years of work experience:

- 1-10
- > 10 years

QUESTIONNAIRE

Section 1: Awareness

1. I am aware of the Primary Health Care Integration (PHCI) program.

   a. Strongly disagree

   b. Disagree

   c. Neutral

   d. Agree

   e. Strongly agree

2. I have frequently received information about the Primary Health Care Integration in the past three months.

   a. Strongly disagree

b. Disagree

c. Neutral

d. Agree

e. Strongly agree

3. Information about PHCI has been well disseminated among health workers and cadres.

a. Strongly disagree

b. Disagree

c. Neutral

d. Agree

e. Strongly agree

Section 2: Comprehension

4. I understand that PHCI is a national policy that must be implemented across Indonesia.

a. Strongly disagree

b. Disagree

c. Neutral

d. Agree

e. Strongly agree

5. I understand that the goal of PHCI is to provide services to people of all ages, from pregnant women to the elderly.

a. Strongly disagree

b. Disagree

c. Neutral

d. Agree

e. Strongly agree

6. I understand that a key change in PHCI is the establishment of a single Health Posts serving all age groups (replacing Integrated Health Monitoring Post, Youth Health Posts, and Elderly Health Posts).

a. Strongly disagree

b. Disagree

c. Neutral

d. Agree

e. Strongly agree

7. I understand that differences between urban and rural areas will affect PHCI implementation at Health Posts.

   a. Strongly disagree

   b. Disagree

   c. Neutral

   d. Agree

   e. Strongly agree

Section 3: Concern

8. I care about the integration of Health Posts, Posbindu, and Youth Health Posts services into a single Health Posts service.

   a. Strongly disagree

   b. Disagree

   c. Neutral

   d. Agree

   e. Strongly agree

9. I care about competency training for health services as part of PHCI at Health Posts.

   a. Strongly disagree

   b. Disagree

   c. Neutral

   d. Agree

   e. Strongly agree

10. I care about the quality of health services through PHCI at Health Posts.

    a. Strongly disagree

    b. Disagree

    c. Neutral

    d. Agree

    e. Strongly agree

11. I care about the successful implementation of PHCI at Health Posts.

    a. Strongly disagree

    b. Disagree

    c. Neutral

    d. Agree

e. Strongly agree

Section 4: Involvement

12. I am willing to be actively involved in the implementation of PHCI at Health Posts.
    a. Strongly disagree
    b. Disagree
    c. Neutral
    d. Agree
    e. Strongly agree

13. I actively participate in activities related to PHCI at Health Posts.

    a. Strongly disagree

    b. Disagree

    c. Neutral

    d. Agree

    e. Strongly agree

14. I am willing to be actively involved in home visit activities as part of PHCI implementation at Health Posts.

    a. Strongly disagree

    b. Disagree

    c. Neutral

    d. Agree

    e. Strongly agree

15. I am willing to be actively involved in reporting community health data as part of PHCI implementation at Health Posts.

    a. Strongly disagree

    b. Disagree

    c. Neutral

    d. Agree

    e. Strongly agree

Section 5: Support

16. Support from the City Health Office is essential for the implementation of PHCI at Health Posts.
    a. Strongly disagree
    b. Disagree
    c. Neutral
    d. Agree
    e. Strongly agree

17. Support from the Subdistrict Office is essential for the implementation of PHCI at Health Posts.
    a. Strongly disagree
    b. Disagree
    c. Neutral
    d. Agree
    e. Strongly agree
18. Support from the Head of Neighbourhood is essential for the implementation of PHCI at Health Posts.
    a. Strongly disagree
    b. Disagree
    c. Neutral
    d. Agree
    e. Strongly agree
19. Support from educational institutions can contribute to the implementation of PHCI at Health Posts.
    a. Strongly disagree
    b. Disagree
    c. Neutral
    d. Agree
    e. Strongly agree

Section 6: Supporting Factors

20. The enthusiasm of health cadres is crucial to supporting the implementation of PHCI at Health Posts.
    a. Strongly disagree
    b. Disagree
    c. Neutral
    d. Agree
    e. Strongly agree
21. Collaboration among all parties is essential for the implementation of PHCI at Health Posts.
    a. Strongly disagree
    b. Disagree
    c. Neutral
    d. Agree
    e. Strongly agree
22. Commitment from all stakeholders is vital for the implementation of PHCI at Health Posts.
    a. Strongly disagree
    b. Disagree
    c. Neutral
    d. Agree
    e. Strongly agree
23. I support the implementation of PHCI that adapts to local conditions for better healthcare service quality.
    a. Strongly disagree

b. Disagree
c. Neutral
d. Agree
e. Strongly agree

Part 7: Barriers

24. The shortage of health cadres significantly hinders the implementation of PHCI at Health Posts.
    a. Strongly disagree
    b. Disagree
    c. Neutral
    d. Agree
    e. Strongly agree

25. Limited healthcare facilities pose a major challenge to the implementation of PHCI.
    a. Strongly disagree
    b. Disagree
    c. Neutral
    d. Agree
    e. Strongly agree

26. In my opinion, health cadres will have difficulties in reporting data online for the implementation of PHCI at Health Posts.
    a. Strongly disagree
    b. Disagree
    c. Neutral
    d. Agree
    e. Strongly agree

Part 8: Readiness

27. The Health Posts in my area are ready to implement PHCI.
    a. Strongly disagree
    b. Disagree
    c. Neutral
    d. Agree
    e. Strongly agree

28. The Health Posts in my area have at least five health cadres, which is one of the requirements for PHCI.
    a. Strongly disagree
    b. Disagree
    c. Neutral
    d. Agree
    e. Strongly agree

29. The health cadres in my area have mastered the 25 basic competencies required for PHCI.
    a. Strongly disagree
    b. Disagree
    c. Neutral

d. Agree
e. Strongly agree

30. The Health Posts in my area are ready to integrate services (merging Health Posts, Posbindu, and Adolescent Health Posts).
a. Strongly disagree
b. Disagree
c. Neutral
d. Agree
e. Strongly agree

31. The Health Posts in my area are prepared to finance home visit activities.
a. Strongly disagree
b. Disagree
c. Neutral
d. Agree
e. Strongly agree

32. The Health Posts in my area have adequate facilities for home visit activities.
a. Strongly disagree
b. Disagree
c. Neutral
d. Agree
e. Strongly agree
