# Supplementary figures and images for: Readiness of health posts for primary health care integration in Indonesia: a mixed-methods study
Source: BMC Public Health. 2025 Apr 16;25:1429. doi: 10.1186/s12889-025-22520-x (PMC12001397; doi:10.1186/s12889-025-22520-x)

# Thematic Framework of Qualitative Findings

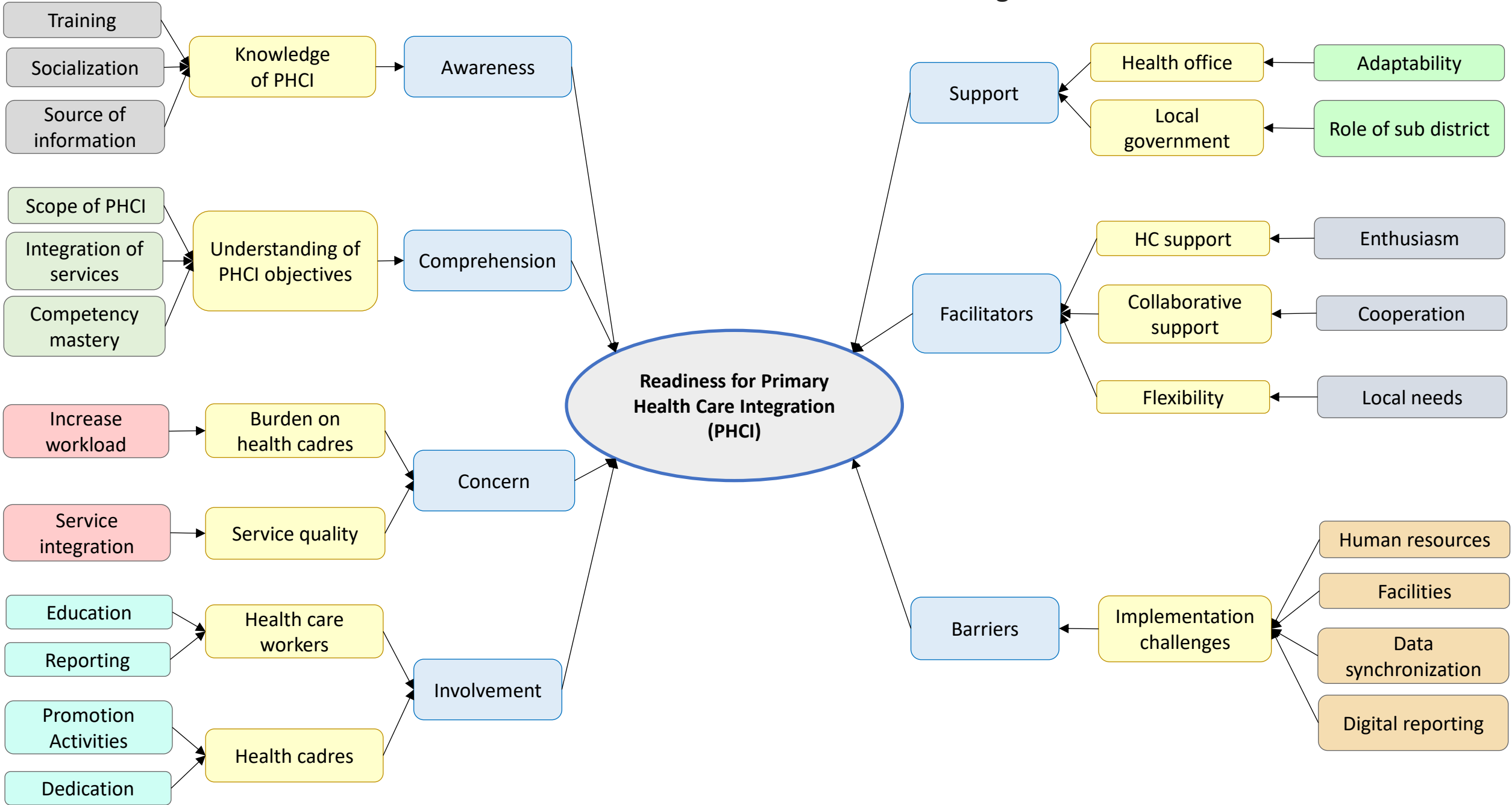

Supplement: Supplementary file 6 — Supplementary Material 6 [file 12889_2025_22520_MOESM6_ESM.pdf]
